# Gi/o GPCRs drive the formation of actin-rich tunneling nanotubes in cancer cells *via* a Gβγ/PKCα/FARP1/Cdc42 axis

**DOI:** 10.1016/j.jbc.2023.104983

**Published:** 2023-06-28

**Authors:** Mariana Cooke, Suli Zhang, Fabiana Cornejo Maciel, Marcelo G. Kazanietz

**Affiliations:** 1 Department of Systems Pharmacology and Translational Therapeutics, Perelman School of Medicine, University of Pennsylvania, Philadelphia, Pennsylvania, USA; 2 Departament of Human Biochemistry, School of Medicine, University of Buenos Aires, Buenos Aires, Argentina; 3 INBIOMED, CONICET, Buenos Aires, Argentina

**Keywords:** tunneling nanotubes, signal transduction, GPCR, eicosanoids, OXER1, 5-oxo-ETE, phospholipase C, PKC, PI3K, small GTPases, Cdc42, FARP1, cancer cells

## Abstract

The functional association between stimulation of G-protein–coupled receptors (GPCRs) by eicosanoids and actin cytoskeleton reorganization remains largely unexplored. Using a model of human adrenocortical cancer cells, here we established that activation of the GPCR OXER1 by its natural agonist, the eicosanoid 5-oxo-eicosatetraenoic acid, leads to the formation of filopodia-like elongated projections connecting adjacent cells, known as tunneling nanotube (TNT)-like structures. This effect is reduced by pertussis toxin and GUE1654, a biased antagonist for the Gβγ pathway downstream of OXER1 activation. We also observed pertussis toxin-dependent TNT biogenesis in response to lysophosphatidic acid, indicative of a general response driven by Gi/o-coupled GPCRs. TNT generation by either 5-oxo-eicosatetraenoic acid or lysophosphatidic acid is partially dependent on the transactivation of the epidermal growth factor receptor and impaired by phosphoinositide 3-kinase inhibition. Subsequent signaling analysis reveals a strict requirement of phospholipase C β3 and its downstream effector protein kinase Cα. Consistent with the established role of Rho small GTPases in the formation of actin-rich projecting structures, we identified the phosphoinositide 3-kinase–regulated guanine nucleotide exchange factor FARP1 as a GPCR effector essential for TNT formation, acting *via* Cdc42. Altogether, our study pioneers a link between Gi/o-coupled GPCRs and TNT development and sheds light into the intricate signaling pathways governing the generation of specialized actin-rich elongated structures in response to bioactive signaling lipids.

Eicosanoids generated from arachidonic acid control diverse physiological and pathological processes through the activation of specific G-protein–coupled receptors (GPCRs) (1, 2, 3). The lipoxygenase branch of arachidonic acid metabolism generates 5-hydroperoxyeicosatetraenoic acid, a precursor for leukotrienes and 5-hydroxy-eicosatetraenoic acid (4). 5-oxo-eicosatetraenoic acid (5-oxo-ETE) derives from 5-hydroxy-eicosatetraenoic acid oxidation and acts as a potent inflammatory mediator and chemoattractant in eosinophils and basophils, playing important roles in the pathophysiology allergic diseases and asthma (5, 6, 7, 8). In addition, 5-oxo-ETE promotes proliferation, survival, and migration in cancer cells and regulates steroidogenesis in adrenocortical cells (9, 10, 11, 12, 13, 14). The actions of 5-oxo-ETE are mediated by OXER1, a GPCR that couples to heterotrimeric Gi/o proteins (8, 9, 10, 15, 16, 17).

Stimulation of GPCRs, including Gi/o-coupled receptors, leads to the activation of Rho small G-proteins (18, 19), a family of GTPases widely implicated in actin cytoskeleton reorganization. RhoA, Cdc42, and Rac1, the three main members of the Rho GTPase family, drive signal platforms for the formation of stress fibers, filopodia, and lamellipodia/ruffles, respectively. The dynamic reorganization of these actin-rich structures represents a fundamental step in the control of cell motility, cell polarity, and cell–cell communication (20, 21, 22, 23). Guanine nucleotide exchange factors (GEFs) facilitate GTP loading and stimulate the activation of Rho G-proteins, while GTPase-activating proteins promote their inactivation (20, 24, 25). The large number of Rho family GEFs and GTPase-activating protein, with distinctive expression patterns, regulatory modes and small G-protein specificity, predicts complex signaling programs designed to dynamically control different cytoskeletal regulatory activities (25, 26, 27, 28, 29, 30). To date, there is scarce information regarding the effects of 5-oxo-ETE on the formation of actin-rich structures driven by Rho GTPase family members.

In this study, we found that OXER1 stimulation promotes the formation of actin-rich tunneling nanotube (TNT)-like structures (31, 32, 33) († see note). A meticulous analysis of downstream OXER1 signaling effectors pinned down the Gβγ–phospholipase C (PLC)–protein kinase C (PKC) axis and the GEF FARP1 as crucial mediators of TNT biogenesis.

## Results

### OXER1 stimulation promotes the formation of TNT-like structures

**Figure 1:**
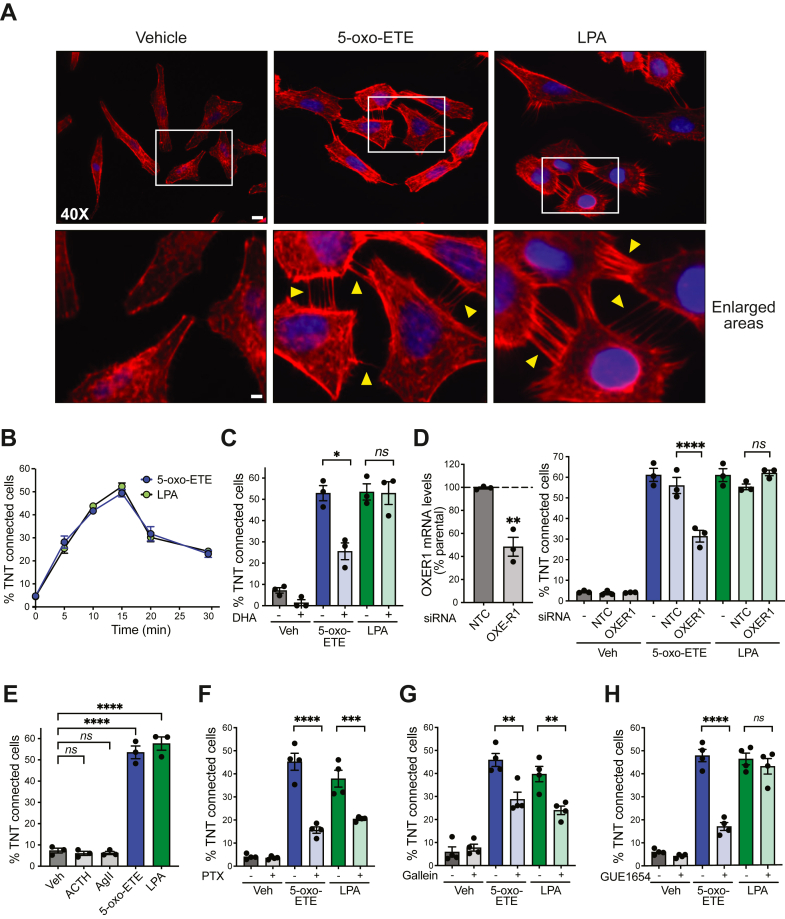
**5-oxo-ETE and LPA induce the formation of TNT-like structures *via* Gβγ subunits.** Serum-starved H295R cells were treated with either 5-oxo-ETE (500 nM) or LPA (100 nM), stained with rhodamine phalloidin, and visualized by fluorescence microscopy. *A*, representative micrographs. Scale bar *upper panels*, 12 μm. Scale bar *lower panels*, 4 μm. *B*, time-course analysis. *C*, effect of DHA (50 μM); (*D*), effect of OXER1 RNAi. OXER1 mRNA expression (*left*); quantitative analysis (*right*). *Dotted line*, parental cells. *E*, lack of effect of 100 nM angiotensin II or 1 nM ACTH (15 min). *F*, effect of PTX (100 ng/ml, 24 h). *G*, effect of gallein (3 μM, 1 h). *H*, effect of GUE1654 (10 μM, 1 h). Results are expressed as mean ± S.E.M. ∗*p* < 0.05, ∗∗*p* < 0.01, ∗∗∗*p* < 0.001, ∗∗∗∗*p* < 0.0001. 5-oxo-ETE, 5-oxo-eicosatetraenoic acid; *Arrows*, TNT-like structures; DHA, docosahexaenoic acid; LPA, lysophosphatidic acid; *ns*, not significant; *NTC*, nontarget control; PTX, pertussis toxin; RNAi, RNA interference; TNT, tunneling nanotube.

In order to study OXER1-mediated effects, we used the H295R human adrenocortical cancer cell line, an established model for 5-oxo-ETE-mediated responses (11, 13, 14). During initial studies, we observed significant changes in H295R cell morphology upon 5-oxo-ETE treatment, namely the formation of spike-like cell surface protrusions. Rhodamine-phalloidin staining for filamentous actin (F-actin) revealed that in addition to thin membrane protrusions resembling filopodia, 5-oxo-ETE–treated cells produced long actin-rich structures connecting distant cells (Fig. 1*A*). The later structures, known as TNT-like structures—hereafter “TNT-like structures”—have been described in numerous cellular models and play important roles in intercellular adhesion and communication (31, 32, 33, 34). TNT-like structures can be readily detected 5 min after the addition of 5-oxo-ETE, with the maximum effect reaching ∼50% of connected cells achieved at 15 min (Fig. 1*B*). The 5-oxo-ETE effect was diminished by pretreatment with the OXER1 antagonist docosahexaenoic acid (Fig. 1*C*) and by OXER1 RNA interference (RNAi)-mediated silencing (Fig. 1*D*). No protrusive or connecting structures could be detected upon inhibition of actin polymerization with either latrunculin A or cytochalasin D (data not shown).

To determine if the observed 5-oxo-ETE effect is produced by other Gi/o ligand, we used lysophosphatidic acid (LPA). Notably, LPA caused a similar time-dependent formation of TNT-like structures in H295R cells. As expected, the LPA effect was insensitive to docosahexaenoic acid or OXER1 silencing (Fig. 1, *A*–*D*). Stimulation of GPCRs coupled to Gs or Gq, namely ACTH and angiotensin II receptors, respectively, failed to induce TNT-like structures (Fig. 1*E*).

### TNT-like structure formation by Gi/o-GPCRs is mediated by Gβγ subunits

As a Gi/o-coupled receptor, OXER1-mediated functions are sensitive to pertussis toxin (16, 17), which inhibits the dissociation of the Gαi-βγ heterotrimeric complex. We found that TNT-like structure formation in response to either 5-oxo-ETE or LPA was markedly reduced in H295R cells subjected to pertussis toxin treatment (Fig. 1*F*). Gallein, a Gβγ inhibitor, caused a similar effect (35) (Fig. 1*G*). Remarkably, a biased inhibitor specific for the OXER1/Gβγ pathway, GUE1654 (36, 37), reduced the development of 5-oxo-ETE-induced TNT-like structures without affecting the LPA response (Fig. 1*H*), which attests to the selectivity of the OXER1 biased inhibitor. Taken together, these results indicate that TNT-like structure development in response to Gi/o ligands depends on Gβγ subunits dissociated from heterotrimeric G proteins. The Gβγ-dependent formation of TNT-like structures by Gi/o ligands was replicated in DU145 cells, a prostate cancer cell line that expresses OXER1 (10) (Fig. S1, *A* and *B*).

### Epidermal growth factor transactivation mediates the development of TNT-like structures by Gi/o GPCR ligands

**Figure 2:**
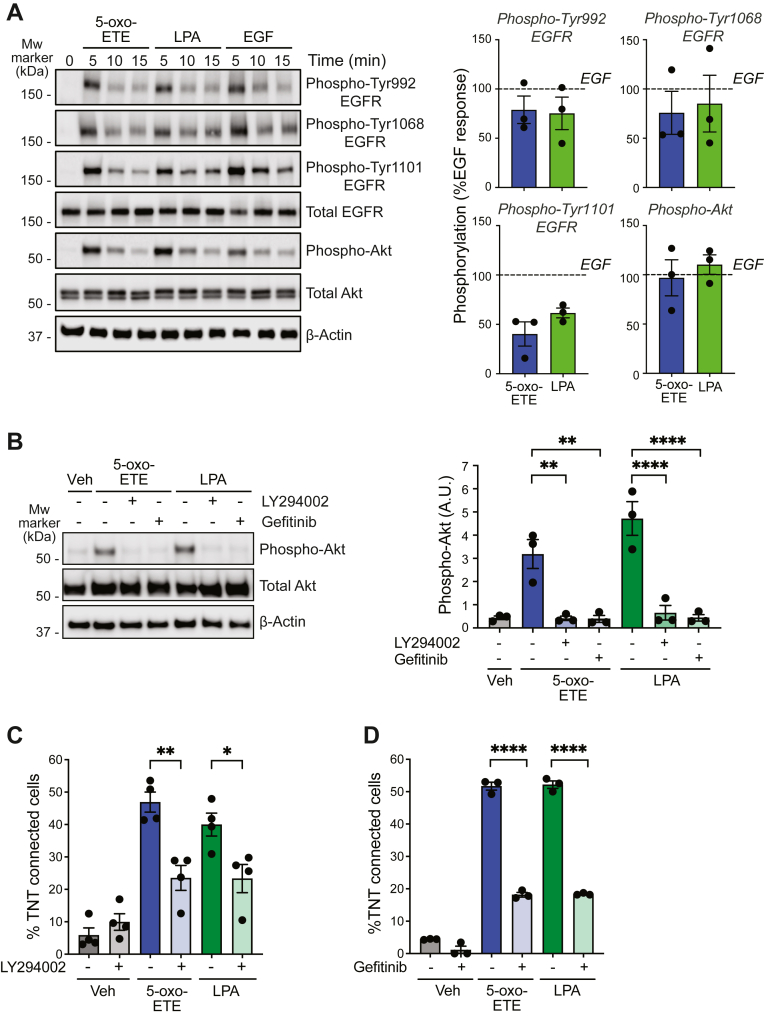
**TNT-like structure formation by GPCR ligands involves EGFR transactivation.***A*, representative Western blot for phospho-EGFR and phospho-Akt in response to 5-oxo-ETE (500 nM), LPA (100 nM), or EGF (200 nM) in H295R cells (*left*). Densitometric analysis (*right*) expressed as % of the EGF effect (*dotted line*). *B*, effect of gefitinib (3 μM, 1 h) and LY294002 (20 μM, 1 h) on Akt activation. Representative experiment (*left*). Densitometric analysis (*right*). *A.U.*, arbitrary units. *C*, effect of LY294002 on TNT formation. *D*, effect of gefitinib on TNT formation. Results are expressed as mean ± S.E.M. *ns*, not significant, ∗*p* < 0.05, ∗∗*p* < 0.01, ∗∗∗∗*p* < 0.0001. 5-oxo-ETE, 5-oxo-eicosatetraenoic acid; EGFR, epidermal growth factor receptor; GPCR, G-protein–coupled receptor; TNT, tunneling nanotube.

GPCR-mediated responses may involve transactivation mechanisms *via* receptor tyrosine kinases such as epidermal growth factor receptor (EGFR) (38). Both 5-oxo-ETE and LPA triggered a rapid phosphorylation of EGFR in Tyr992, Tyr1068, and Tyr1101 residues. Likewise, both ligands caused strong phosphorylation (*i.e.*, activation) of Akt, a well-established phosphoinositide 3-kinase (PI3K) effector, with similar kinetics to EGFR phosphorylation (Fig. 2*A*). Akt activation by these GPCR ligands was impaired by the PI3K inhibitor LY294002. Most remarkably, Akt activation by either ligand was abolished by the specific EGFR inhibitor gefitinib (Fig. 2*B*), suggesting that activation of the PI3K pathway proceeds *via* a GPCR–EGFR transactivation mechanism. Both 5-oxo-ETE- and LPA-induced generation of TNT-like structures was markedly diminished by LY294002 (Fig. 2*C*), indicating the requirement of the PI3K pathway in this response. Interestingly, gefitinib caused a prominent reduction in the formation of TNT-like structures by 5-oxo-ETE or LPA in adrenocortical cells (Fig. 2*D*). TNT-like structures could not be detected in response to EGF (data not shown), arguing that EGFR activation, while required for the effect of Gi/o-GPCR ligands, was insufficient to trigger this morphological change. We also observed that TNT formation by 5-oxo-ETE was not affected by the MEK inhibitor PD98059 (% TNT connected cells: - PD98058 54.0 ± 3.8; + 20 μM PD98059: 54.3 ± 2.9, not significant). This suggests that the ERK pathway, although becoming activated by OXER1 stimulation (11), was dispensable for TNT formation. The Gi/o GPCR–EGFR transactivation and the EGFR/PI3K dependency for 5-oxo-ETE-induced TNT formation were also evident in DU145 prostate cancer cells (Figs. S1*B* and S2).

### Requirement of the PLC-PKC pathway in TNT-like structure formation

**Figure 3:**
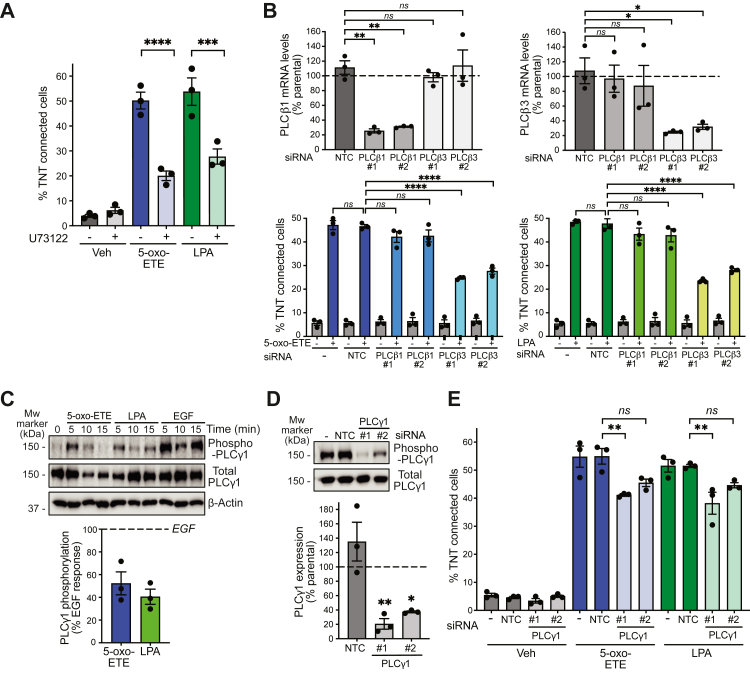
**PLC dependency of TNT-like structure formation.***A*, effect of U73122 (10 μM, 1 h) on TNT formation by 5-oxo-ETE and LPA in H295R cells. *B*, PLCβ1 and PLCβ3 RNAi. mRNA levels were determined by Q-PCR (*upper*). *Dotted line*, parental cells. Quantitation of TNT-like structure formation (*lower*). *C*, representative Western blot (*upper*) and densitometric analysis for phospho-PLCγ1 (*lower*), expressed as % of the EGF response (*dotted line*). *D*, PLCγ1 RNAi. Representative experiment (*upper*); densitometric analysis (*lower*). *Dotted line*, parental cells. *E*, TNT-like structure formation in PLCγ1-depleted cells (mean ± S.E.M.). ∗*p* < 0.05, ∗∗*p* < 0.01, ∗∗∗*p* < 0.001, ∗∗∗∗*p* < 0.0001. 5-oxo-ETE, 5-oxo-eicosatetraenoic acid; LPA, lysophosphatidic acid; *ns*, not significant; *NTC*, nontarget control; PLC, phospholipase C; Q-PCR, quantitative polymerase chain reaction; RNAi, RNA interference; TNT, tunneling nanotube.

5-oxo-ETE can elevate intracellular Ca^2+^ levels, likely *via* utilization of PLC (10, 16), a family of enzymes responsible for Ca^2+^ mobilization and generation of the lipid second messenger diacylglycerol (DAG). While it is well recognized that GPCRs couple to PLCβ *via* Gαq subunits (39), there is no evidence that OXER1 couples to the heterotrimeric Gq proteins. Nonetheless, Gβγ subunits released upon stimulation of Gi/o-coupled GPCRs bind to and activate PLCβ. Moreover, Gi/o GPCR ligands raise intracellular Ca^2+^ and promote the activation of PKC, the main DAG effector (39, 40, 41, 42). This led us to speculate that OXER1-mediated TNT-like structure formation may involve the PLCβ-PKC pathway. Figure 3*A* shows that the PLC inhibitor U73122 reduced the formation of TNT-like structures by either 5-oxo-ETE or LPA in H295R cells.

We next used RNAi to silence PLCβ1 and PLCβ3, the PLCβ-isozymes predominantly expressed in cancer cells (39, 41), achieving ∼75 to 80% knockdown in each case using two different small-interfering RNA (siRNA) duplexes (Fig. 3*B*, *upper panels*). These studies revealed a PLCβ3-dependency for the biogenesis of TNT-like structures in H295R cells, both in response to either 5-oxo-ETE or LPA, whereas PLCβ1 was dispensable (Fig. 3*B*, *lower panels*).

Based on the identified Gi/o-coupled GPCR/EGFR transactivation and considering that EGFR couples to PLCγ1 (43, 44), we also examined a potential implication of this PLC isoform. Both 5-oxo-ETE and LPA induced the phosphorylation of PLCγ1 in Tyr783, a hallmark of PLCγ1 activation, although the effect was lower than that caused by EGF treatment (Fig. 3*C*). In H295R cells subjected to PLCγ1 RNAi (Fig. 3*D*), we observed a slight inhibition (∼20%) in TNT-like structure formation by both 5-oxo-ETE and LPA, although statistical significance was only achieved with one PLCγ1 siRNA duplex (Fig. 3*E*). Taken together, these experiments suggest that in H295R cells the PLC input leading to TNT-like structure formation arises predominantly from PLCβ3.

**Figure 4:**
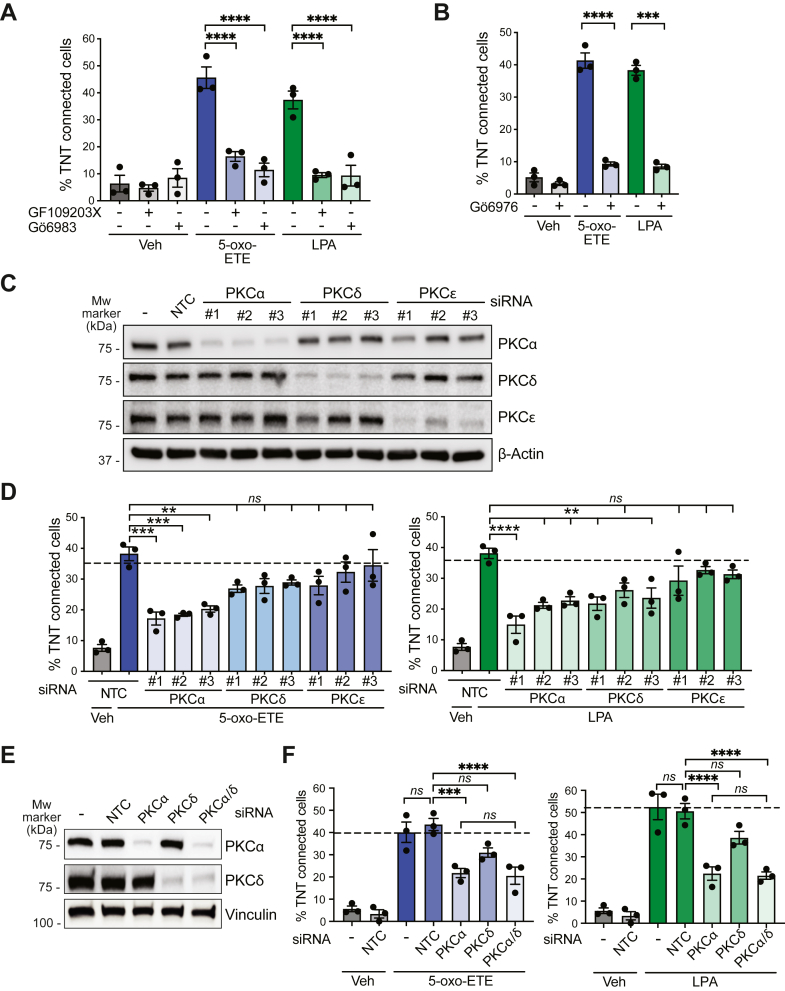
**PKC isozyme dependency for the formation of TNT-like structures.***A*, effect of GF109203X and Gö6983 (5 μM) on TNT formation by 5-oxo-ETE (500 nM, 15 min) and LPA (100 nM, 15 min) in H295R cells 1 h). *B*, effect of Gö6976 (5 μM, 1 h). *C*, representative Western blot for RNAi-mediated depletion of PKC isozymes. *D*, TNT formation upon individual PKC isozyme RNAi. *E*, Western blot for dual PKCα/PKCδ RNAi depletion. *F*, TNT formation upon dual PKCα/PKCδ RNAi. Results are expressed as mean ± S.E.M. *Dotted line*, parental cells. *NTC*, nontarget control. *ns*, not significant, ∗∗*p* < 0.01, ∗∗∗*p* < 0.001, ∗∗∗∗*p* < 0.0001. 5-oxo-ETE, 5-oxo-eicosatetraenoic acid; LPA, lysophosphatidic acid; PKC, protein kinase C; RNAi, RNA interference; TNT, tunneling nanotube.

Next, we examined whether Gi/o coupled GPCR-mediated TNT-like structure formation involves PKC. There are seven PKC family members responsive to DAG (41), four of them being Ca^2+^-dependent (“classical/conventional” PKCs α, β, and γ) and three Ca^2+^ insensitive (“novel” PKCs δ, ε, η, and θ). Pretreatment with the “pan” PKC inhibitors GF109203X or Gö6983 (Fig. 4*A*), or with the classical/conventional PKC inhibitor Gö6976, abrogated the formation of TNT-like structures in response Gi/o GPCR ligands both in H295R cells (Fig. 4*B*) and DU145 cells (Fig. S1*C*). RNAi-specific depletion of PKCα, PKCδ, and PKCε (Fig. 4*C*), the PKC isozymes mainly expressed in H295R cells, unveiled PKCα as the main PKC involved in the formation of TNT-like structures by both ligands (Fig. 4*D*). Upon PKCδ depletion, a slight, still statistically significant inhibition could be detected in response to LPA but not to 5-oxo-ETE, although not with all three RNAi duplexes. PKCε RNAi had no effect on the response by the Gi/o coupled-GPCR ligands. There were no additive effects by dual knockdown of PKCα and PKCδ (Fig. 4, *E* and *F*), pointing to PKCα as the main PKC implicated in Gi/o-coupled GPCR-mediated generation of TNT-like structures.

### The Rho-GEF FARP1 mediates TNT-like structure formation

**Figure 5:**
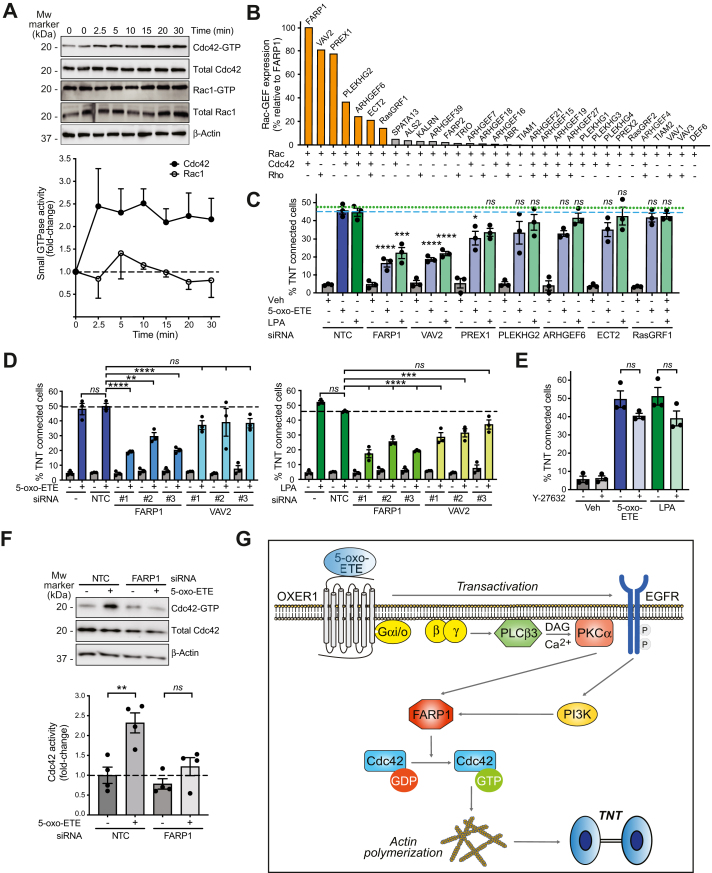
**FARP1 requirement for TNT-like structure generation.***A*, representative Cdc42 and Rac1 activation assays, using 500 nM 5-oxo-ETE (*upper*), and densitometric analysis, normalized to loading control (n = 3–6). *Dotted line*, nonstimulated cells (*lower*). *B*, expression of Rho family GEFs in H295R cells (Q-PCR array, normalized to FARP1). The known activity of each GEF on Rac, Cdc42, and Rho is shown. *Orange*, GEFs selected for functional analysis. *C*, effect of GEF RNAi on TNT-like structure formation. *Dotted blue line*, parental cells (5-oxo-ETE). *Pointed green line*, parental cells (LPA). *D*, TNT-like structure formation in FARP1 and VAV2 depleted cells. *E*, effect of Y-27632. *F*, representative Cdc42 pull-down assay in FARP1-silenced cells (*upper*) and densitometric analysis (*lower*). *NTC*, nontarget control. *ns*, not significant, ∗*p* < 0.05, ∗∗*p* < 0.01, ∗∗∗*p* < 0.001, ∗∗∗∗*p* < 0.0001. *G*, hypothetical model for the formation of TNT-like structures by Gi/o GPCR ligands. 5-oxo-ETE, 5-oxo-eicosatetraenoic acid; GEF, Guanine nucleotide exchange factor; GPCR, G-protein–coupled receptor; Q-PCR, quantitative polymerase chain reaction; RNAi, RNA interference; TNT, tunneling nanotube.

Although there is limited information connecting Rho family GTPases to TNT-like structure generation (22, 45), a study done in macrophages points to Cdc42 as the predominant Rho GTPase involved in this response (46). Using “pull-down” assay, we found that 5-oxo-ETE caused a significant rise in Cdc42 levels in H295R cells, whereas no activation of Rac1 could be detected (Fig. 5*A*).

Activation of Rho GTPases is mediated by GEFs, whose activity is in most cases dependent on PI3K (23, 47). To determine GEF expression, we took advantage of a predesigned Rac-GEF quantitative polymerase chain reaction (Q-PCR) array that includes multiple members displaying activity on Cdc42 (29) (Fig. 5*B*). This analysis revealed FARP1—a Rac/Cdc42 exchange factor (29, 48, 49)—as the main GEF expressed in H295R cells. Silencing the expression of the seven top expressed GEF candidates (Fig. S3*A*) identified a strong FARP1 dependency for TNT-like structure biogenesis both in response to 5-oxo-ETE and LPA, followed by VAV2. Other GEFs highly expressed in H295R cells (*i.e.*, PREX1, PLEKHG2, ARHGEF6, ECT2, RASGRF1) were mostly dispensable (Fig. 5*C*). The FARP1 requirement was validated using three different RNAi duplexes (silencing shown in Fig. S3*B*). A lesser inhibitory effect was observed for VAV2 RNAi only upon LPA stimulation, which reached statistical significance with only two out of three siRNA duplexes used (Fig. 5*D*). The Rho kinase inhibitor Y-27632 had no effect, suggesting that Rho signaling is dispensable (Fig. 5*E*).

Finally, we observed that Cdc42 activation by 5-oxo-ETE was significantly inhibited in H295R cells subjected to FARP1 RNAi (Fig. 5*F*). Altogether, our results establish FARP1 as a key Gi/o GPCR effector for TNT-like structures formation, acting *via* Cdc42.

## Discussion

The main conceptual finding from our study is that cell surface receptors—specifically Gi/o-coupled GPCRs—drive the formation of TNT-like structures, thus providing the first evidence for a link between extracellular signals and TNT generation. TNTs are submicrometer thin connecting structures that play important roles in cell–cell communication both in development and disease progression (31, 32, 33). Although TNTs have been initially defined as open-ended channels for cellular material transfer, recent evidence indicates that a vast majority are close-ended and connect paired cell bodies through cadherin adhesion molecules (34). While the *de novo* events involved in TNT formation are yet to be disentangled, ultrastructural imaging approaches suggest that they primarily derive from thin protrusive filopodia structures (34, 50). Identifying a Gβγ-FARP1-Cdc42 axis underscores a novel signaling mechanism downstream of Gi/o-coupled GPCRs impinging upon actin cytoskeleton reorganization while providing a framework for follow-up mechanistic analysis of TNT biogenesis. The Gβγ subunit requirement for TNT-like structure development aligns with prior 5-oxo-ETE chemoattraction studies in eosinophils and neutrophils (37).

Our findings denote a role for PKC, mainly PKCα, in TNT formation. Other DAG-dependent PKCs remain mostly dispensable for TNT formation by stimulation of Gi/o-coupled GPCRs, despite becoming activated (11). Previous studies demonstrated the involvement of PKCs (including PKCα) in Cdc42 activation and filopodia development (51, 52, 53). Although the mechanisms regulating FARP1 activation are not yet understood, an appealing scenario is that PKCα—or a PKCα downstream kinase—directly phosphorylates FARP1 to promote its Cdc42 exchange activity. Indeed, Rho family GEFs are subjected to direct phosphorylation and autoinhibition relief (23, 47). A preliminary analysis using NetPhos 3.1 predicts high-scoring PKC phosphorylation sites in FARP1, both in the N-terminal FERM domain driving membrane association and the C-terminal autoinhibitory site (data not shown). Additionally, PKCα associates with fascin, an actin-bundling component of filopodia (54, 55), and phosphorylates Cdc42 effector protein-4, an event that facilitates filopodia formation (52). PKCα also regulates the function of N-cadherin (56), an adhesion molecule that accumulates at the junction between the filopodium and the cell body of the connecting cell (34), thus providing a mechanistic foundation for the stabilization of TNT-like structures. It would be interesting to analyze whether PKCα-regulatory proteins associated with small GTPase function and cytoskeletal reorganization, such as the proteoglycan syndecan-4 (57), play any role in TNT formation in response to GPCR ligands.

In our adrenocortical human model, the main source for PKC activation arises from PLCβ3. Although we cannot completely rule out the involvement of the canonical Gαq-PLCβ axis—a powerful input for the elevation in DAG/calcium levels and PKCα activation—, this is unlikely based on the known coupling mechanisms for OXER1 (58). While we found a limited contribution of PLCγ1 to the Gi/o GPCR ligand response, our results unambiguously indicate the GPCR-EGFR transactivation as an essential event for PI3K pathway activation. EGFR coupling to PI3K is primarily mediated by the Gab1 adaptor *via* the phosphorylation of Tyr1068 in EGFR (59), a site that is heavily phosphorylated in response to both 5-oxo-ETE and LPA. Activation of PI3K may also occur *via* direct activation by Gβγ subunits (18, 60). Consistent with these observations, FARP1 activation depends on PI3K-derived phosphoinositide products *via* its pleckstrin homology and/or FERM domains (61). Altogether, FARP1/Cdc42-dependent TNT-like structure formation engages multiple inputs stemming from both Gβγ subunits and EGFR transactivation, as summarized in the model presented in Figure 5*G*. Due to the large number of Rho-GEF family members encoded by the genome and their cell type–specific distribution (23, 27, 28, 29, 47, 62), it is plausible that the GPCR/GEF/Cdc42 axis varies depending upon the cellular context. It is worth mentioning that the small GTPase Rac can also play a role in TNT assembly to some extent (22, 45, 46). However, no Rac1 activation by OXER1 stimulation could be observed in our model. Regardless, any potential participation of Rac1 may strictly depend on FARP1—a dual Rac/Cdc42-GEF (29, 48, 49)—since our screening revealed that other Rac-GEFs are either poorly expressed or not required for TNT-like structure formation.

In summary, we identified a signaling paradigm leading to the formation of TNT-like structures *via* the activation of Gi/o-coupled GPCRs. Particularly in cancer cells, TNT-like structures have been linked to proliferation, angiogenesis, invasion, and survival. They have been associated with the transfer of oncogenic molecules (such as mutant KRAS), bioenergetics plasticity, and drug resistance (22, 33, 45, 63, 64, 65, 66). Aberrant oncogenic inputs may influence the formation of these intercellular connecting structures, a paradigm described for other distant cell-to-cell communication systems (*e.g.*, exosomes) (67). TNT development is also sensitive to cellular stress, oxidative damage and ionizing radiation (64, 65, 66), suggesting they are components of an adaptive response to varied stimuli. While the biology of TNT-like structures, either “open-ended” or “close-ended”, embodies an area of intense investigation (32, 33, 34), understanding the signaling mechanisms leading to their formation should contribute to unveil their roles in both physiological and pathological conditions. Although challenging, dissecting the machinery behind actin-rich structure reorganization would aid the development of novel tools, ultimately upstaging the arsenal of disease-related therapeutic approaches.

## Experimental procedures

### Cell culture and reagents

H295R cells (ATCC) were cultured in DMEM-F12 with 10% calf serum and ITS+ Premix (Corning). DU145 cells (ATCC) were cultured in RPMI with 10% fetal bovine serum. Reagents, including siRNA duplexes and antibodies, are listed in Table S1. Treatments were done after 24 h serum starvation. Inhibitors were added for 1 h before and during treatment with different ligands.

### RNA interference

siRNA duplexes (30 nM) were transfected using Lipofectamine RNAi Max (Invitrogen). Experiments were carried out 48 h after transfection.

### Western blot and Q-PCR assay

Western blots were performed essentially as described previously (68). Bands visualization and densitometric analysis were done with an Odyssey Fc system (LI-COR Biotechnology).

Q-PCR was done as previously described (69). Results were expressed as ΔCt, calculated as the difference between Ct values for each gene and the UBC housekeeping gene.

### Rhodamine-phalloidin staining

F-actin staining with rhodamine phalloidin was described elsewhere (70). Cells were visualized using a Nikon TE2000-U fluorescent microscope. For quantitative analysis of TNT-connected cells, 6 to 8 fields were scored in a blindly manner.

### PBD pull-down assays

To determine Cdc42 and Rac1 activation, we used a PBD pulldown assay (29, 71). Precipitated Cdc42-GTP and Rac1-GTP were determined by Western blot using anti-Cdc42 and anti-Rac1 specific antibodies, respectively.

### Statistical analysis

Statistical significance was determined by Student's *t* test or ANOVA using GraphPad Prism version 9.5.1.

## Data availability

All data are contained within the article and Supporting information.

## Supporting information

This article contains supporting information.

## Conflict of interest

The authors declare that they have no conflicts of interest with the contents of this article.

## Footnotes

† Note: While tunneling nanotubes (TNTs) were originally defined as *“open-ended channels mediating membrane continuity between connected cells”*, subsequent studies established that they could be either “open” or “closed”, and therefore they do not necessarily imply transfer capacity (33, 34). Considering the heterogeneity of these connecting structures, for the present study, we will comply with the recommended use of the general term “TNT-like structures” (32, 33).

## References

[1] Samuelsson B. (2012). Role of basic science in the development of new medicines: examples from the eicosanoid field. J. Biol. Chem..

[2] Wang D., Dubois R.N. (2010). Eicosanoids and cancer. Nat. Rev. Cancer.

[3] Im D.S. (2009). New intercellular lipid mediators and their GPCRs: an update. Prostaglandins Other Lipid Mediat..

[4] Brash A.R. (1999). Lipoxygenases: occurrence, functions, catalysis, and acquisition of substrate. J. Biol. Chem..

[5] Powell W.S., Gravel S., MacLeod R.J., Mills E., Hashefi M. (1993). Stimulation of human neutrophils by 5-oxo-6,8,11,14-eicosatetraenoic acid by a mechanism independent of the leukotriene B4 receptor. J. Biol. Chem..

[6] Powell W.S., Rokach J. (2005). Biochemistry, biology and chemistry of the 5-lipoxygenase product 5-oxo-ETE. Prog. Lipid Res..

[7] Powell W.S. (2021). Eicosanoid receptors as therapeutic targets for asthma. Clin. Sci. (Lond.).

[8] Cossette C., Miller L.A., Ye Q., Chourey S., Reddy C.N., Rokach J. (2022). Targeting the OXE receptor with a selective antagonist inhibits allergen-induced pulmonary inflammation in non-human primates. Br. J. Pharmacol..

[9] O'Flaherty J.T., Rogers L.C., Paumi C.M., Hantgan R.R., Thomas L.R., Clay C.E. (2005). 5-Oxo-ETE analogs and the proliferation of cancer cells. Biochim. Biophys. Acta.

[10] Sarveswaran S., Ghosh J. (2013). OXER1, a G protein-coupled oxoeicosatetraenoid receptor, mediates the survival-promoting effects of arachidonate 5-lipoxygenase in prostate cancer cells. Cancer Lett..

[11] Neuman I., Cooke M., Lemiña N.A., Kazanietz M.G., Cornejo Maciel F. (2019). 5-oxo-ETE activates migration of H295R adrenocortical cells via MAPK and PKC pathways. Prostaglandins Other Lipid Mediat..

[12] Kalyvianaki K., Drosou I., Notas G., Castanas E., Kampa M. (2021). Enhanced OXER1 expression is indispensable for human cancer cell migration. Biochem. Biophys. Res. Commun..

[13] Dattilo M., Neuman I., Muñoz M., Maloberti P., Cornejo Maciel F. (2015). OxeR1 regulates angiotensin II and cAMP-stimulated steroid production in human H295R adrenocortical cells. Mol. Cell. Endocrinol..

[14] Cooke M., Di Cónsoli H., Maloberti P., Cornejo Maciel F. (2013). Expression and function of OXE receptor, an eicosanoid receptor, in steroidogenic cells. Mol. Cell. Endocrinol..

[15] Grant G.E., Rokach J., Powell W.S. (2009). 5-Oxo-ETE and the OXE receptor. Prostaglandins Other Lipid Mediat..

[16] Hosoi T., Sugikawa E., Chikada A., Koguchi Y., Ohnuki T. (2005). TG1019/OXE, a Galpha(i/o)-protein-coupled receptor, mediates 5-oxo-eicosatetraenoic acid-induced chemotaxis. Biochem. Biophys. Res. Commun..

[17] Sturm G.J., Schuligoi R., Sturm E.M., Royer J.F., Lang-Loidolt D., Stammberger H. (2005). 5-Oxo-6,8,11,14-eicosatetraenoic acid is a potent chemoattractant for human basophils. J. Allergy Clin. Immunol..

[18] O'Hayre M., Degese M.S., Gutkind J.S. (2014). Novel insights into G protein and G protein-coupled receptor signaling in cancer. Curr. Opin. Cell Biol..

[19] Villaseca S., Romero G., Ruiz M.J., Pérez C., Leal J.I., Tovar L.M. (2022). Gαi protein subunit: a step toward understanding its non-canonical mechanisms. Front. Cell Dev. Biol..

[20] Tapon N., Hall A. (1997). Rho, Rac and Cdc42 GTPases regulate the organization of the actin cytoskeleton. Curr. Opin. Cell Biol..

[21] Ridley A.J. (2015). Rho GTPase signalling in cell migration. Curr. Opin. Cell Biol..

[22] Zhang S., Kazanietz M.G., Cooke M. (2020). Rho GTPases and the emerging role of tunneling nanotubes in physiology and disease. Am. J. Physiol. Cell Physiol..

[23] Cooke M., Baker M.J., Kazanietz M.G. (2020). Rac-GEF/Rac signaling and metastatic dissemination in lung cancer. Front. Cell Dev. Biol..

[24] Kazanietz M.G., Caloca M.J. (2017). The Rac GTPase in cancer: from old concepts to new paradigms. Cancer Res..

[25] Kazanietz M.G., Cooke M., Garcia-Mata R. (2022). Nonredundant Rac-GEF control of actin cytoskeleton reorganization. Trends Cell Biol..

[26] Bustelo X.R. (2018). RHO GTPases in cancer: known facts, open questions, and therapeutic challenges. Biochem. Soc. Trans..

[27] Bagci H., Sriskandarajah N., Robert A., Boulais J., Elkholi I.E., Tran V. (2020). Mapping the proximity interaction network of the Rho-family GTPases reveals signalling pathways and regulatory mechanisms. Nat. Cell Biol..

[28] Müller P.M., Rademacher J., Bagshaw R.D., Wortmann C., Barth C., van Unen J. (2020). Systems analysis of RhoGEF and RhoGAP regulatory proteins reveals spatially organized RAC1 signalling from integrin adhesions. Nat. Cell Biol..

[29] Cooke M., Kreider-Letterman G., Baker M.J., Zhang S., Sullivan N.T., Eruslanov E. (2021). FARP1, ARHGEF39, and TIAM2 are essential receptor tyrosine kinase effectors for Rac1-dependent cell motility in human lung adenocarcinoma. Cell Rep..

[30] Kreider-Letterman G., Carr N.M., Garcia-Mata R. (2022). Fixing the GAP: the role of RhoGAPs in cancer. Eur. J. Cell Biol..

[31] Rustom A., Saffrich R., Markovic I., Walther P., Gerdes H.H. (2004). Nanotubular highways for intercellular organelle transport. Science.

[32] Zurzolo C. (2021). Tunneling nanotubes: reshaping connectivity. Curr. Opin. Cell Biol..

[33] Cordero Cervantes D., Zurzolo C. (2021). Peering into tunneling nanotubes-the path forward. EMBO J..

[34] Chang M., Lee O.C., Bu G., Oh J., Yunn N.O., Ryu S.H. (2022). Formation of cellular close-ended tunneling nanotubes through mechanical deformation. Sci. Adv..

[35] Seneviratne A.M., Burroughs M., Giralt E., Smrcka A.V. (2011). Direct-reversible binding of small molecules to G protein βγ subunits. Biochim. Biophys. Acta.

[36] Blättermann S., Peters L., Ottersbach P.A., Bock A., Konya V., Weaver C.D. (2012). A biased ligand for OXE-R uncouples Gα and Gβγ signaling within a heterotrimer. Nat. Chem. Biol..

[37] Konya V., Blättermann S., Jandl K., Platzer W., Ottersbach P.A., Marsche G. (2014). A biased non-Gαi OXE-R antagonist demonstrates that Gαi protein subunit is not directly involved in neutrophil, eosinophil, and monocyte activation by 5-oxo-ETE. J. Immunol..

[38] Wang Z. (2016). Transactivation of epidermal growth factor receptor by G protein-coupled receptors: recent progress, challenges and future research. Int. J. Mol. Sci..

[39] Katan M., Cockcroft S. (2020). Phospholipase C families: common themes and versatility in physiology and pathology. Prog. Lipid Res..

[40] Fisher I.J., Jenkins M.L., Tall G.G., Burke J.E., Smrcka A.V. (2020). Activation of phospholipase C β by Gβγ and Gαq involves C-terminal rearrangement to release autoinhibition. Structure.

[41] Cooke M., Kazanietz M.G. (2022). Overarching roles of diacylglycerol signaling in cancer development and antitumor immunity. Sci. Signal..

[42] Pfeil E.M., Brands J., Merten N., Vögtle T., Vescovo M., Rick U. (2020). Heterotrimeric G protein subunit Gαq is a master switch for Gβγ-mediated calcium mobilization by Gi-coupled GPCRs. Mol. Cell.

[43] Chattopadhyay A., Vecchi M., Ji Q., Mernaugh R., Carpenter G. (1999). The role of individual SH2 domains in mediating association of phospholipase C-gamma1 with the activated EGF receptor. J. Biol. Chem..

[44] Jang H.J., Suh P.G., Lee Y.J., Shin K.J., Cocco L., Chae Y.C. (2018). PLCγ1: potential arbitrator of cancer progression. Adv. Biol. Regul..

[45] Raghavan A., Rao P., Neuzil J., Pountney D.L., Nath S. (2021). Oxidative stress and Rho GTPases in the biogenesis of tunnelling nanotubes: implications in disease and therapy. Cell. Mol. Life Sci..

[46] Hanna S.J., McCoy-Simandle K., Miskolci V., Guo P., Cammer M., Hodgson L. (2017). The role of Rho-GTPases and actin polymerization during macrophage tunneling nanotube biogenesis. Sci. Rep..

[47] Rossman K.L., Der C.J., Sondek J. (2005). GEF means go: turning on RHO GTPases with guanine nucleotide-exchange factors. Nat. Rev. Mol. Cell Biol..

[48] Amado-Azevedo J., Reinhard N.R., van Bezu J., de Menezes R.X., van Beusechem V.W., van Nieuw Amerongen G.P. (2017). A CDC42-centered signaling unit is a dominant positive regulator of endothelial integrity. Sci. Rep..

[49] Croisé P., Houy S., Gand M., Lanoix J., Calco V., Tóth P. (2016). Cdc42 and Rac1 activity is reduced in human pheochromocytoma and correlates with FARP1 and ARHGEF1 expression. Endocr. Relat. Cancer.

[50] Hase K., Kimura S., Takatsu H., Ohmae M., Kawano S., Kitamura H. (2009). M-Sec promotes membrane nanotube formation by interacting with Ral and the exocyst complex. Nat. Cell Biol..

[51] Shigeta M., Sanzen N., Ozawa M., Gu J., Hasegawa H., Sekiguchi K. (2003). CD151 regulates epithelial cell-cell adhesion through PKC- and Cdc42-dependent actin cytoskeletal reorganization. J. Cell Biol..

[52] Zhao X., Rotenberg S.A. (2014). Phosphorylation of Cdc42 effector protein-4 (CEP4) by protein kinase C promotes motility of human breast cells. J. Biol. Chem..

[53] Sasaki S., Takahashi R., Luo Y., Chujo K., Sera T., Kudo S. (2021). Spatiotemporal distribution of PKCα, Cdc42, and Rac1 before directed cell migration. Biochem. Biophys. Res. Commun..

[54] Heckman C.A., Pandey P., Cayer M.L., Biswas T., Zhang Z.Y., Boudreau N.S. (2017). The tumor promoter-activated protein kinase Cs are a system for regulating filopodia. Cytoskeleton.

[55] Anilkumar N., Parsons M., Monk R., Ng T., Adams J.C. (2003). Interaction of fascin and protein kinase Calpha: a novel intersection in cell adhesion and motility. EMBO J..

[56] Kohutek Z.A., diPierro C.G., Redpath G.T., Hussaini I.M. (2009). ADAM-10-mediated N-cadherin cleavage is protein kinase C-alpha dependent and promotes glioblastoma cell migration. J. Neurosci..

[57] Keller-Pinter A., Gyulai-Nagy S., Becsky D., Dux L., Rovo L. (2021). Syndecan-4 in tumor cell motility. Cancers (Basel).

[58] O'Flaherty J.T., Taylor J.S., Kuroki M. (2000). The coupling of 5-oxo-eicosanoid receptors to heterotrimeric G proteins. J. Immunol..

[59] Rodrigues G.A., Falasca M., Zhang Z., Ong S.H., Schlessinger J. (2000). A novel positive feedback loop mediated by the docking protein Gab1 and phosphatidylinositol 3-kinase in epidermal growth factor receptor signaling. Mol. Cell. Biol..

[60] Maier U., Babich A., Macrez N., Leopoldt D., Gierschik P., Illenberger D. (2000). Gbeta 5gamma 2 is a highly selective activator of phospholipid-dependent enzymes. J. Biol. Chem..

[61] Kuo Y.C., He X., Coleman A.J., Chen Y.J., Dasari P., Liou J. (2018). Structural analyses of FERM domain-mediated membrane localization of FARP1. Sci. Rep..

[62] Casado-Medrano V., Baker M.J., Lopez-Haber C., Cooke M., Wang S., Caloca M.J. (2018). The role of Rac in tumor susceptibility and disease progression: from biochemistry to the clinic. Biochem. Soc. Trans..

[63] Desir S., Wong P., Turbyville T., Chen D., Shetty M., Clark C. (2019). Intercellular transfer of oncogenic KRAS via tunneling nanotubes introduces intracellular mutational heterogeneity in colon cancer cells. Cancers (Basel).

[64] Kretschmer A., Zhang F., Somasekharan S.P., Tse C., Leachman L., Gleave A. (2019). Stress-induced tunneling nanotubes support treatment adaptation in prostate cancer. Sci. Rep..

[65] Valdebenito S., Audia A., Bhat K.P.L., Okafo G., Eugenin E.A. (2020). Tunneling nanotubes mediate adaptation of glioblastoma cells to temozolomide and ionizing radiation treatment. iScience.

[66] Matejka N., Reindl J. (2019). Perspectives of cellular communication through tunneling nanotubes in cancer cells and the connection to radiation effects. Radiat. Oncol..

[67] Xu R., Rai A., Chen M., Suwakulsiri W., Greening D.W., Simpson R.J. (2018). Extracellular vesicles in cancer - implications for future improvements in cancer care. Nat. Rev. Clin. Oncol..

[68] Cooke M., Zhang X., Zhang S., Eruslanov E., Lal P., Daniel R.E. (2022). Protein kinase C alpha is a central node for tumorigenic transcriptional networks in human prostate cancer. Cancer Res. Commun..

[69] Cooke M., Casado-Medrano V., Ann J., Lee J., Blumberg P.M., Abba M.C. (2019). Differential regulation of gene expression in lung cancer cells by diacyglycerol-lactones and a phorbol ester via selective activation of protein kinase C isozymes. Sci. Rep..

[70] Cooke M., Zhou X., Casado-Medrano V., Lopez-Haber C., Baker M.J., Garg R. (2018). Characterization of AJH-836, a diacylglycerol-lactone with selectivity for novel PKC isozymes. J. Biol. Chem..

[71] Baker M.J., Abba M.C., Garcia-Mata R., Kazanietz M.G. (2020). P-REX1-independent, calcium-dependent RAC1 hyperactivation in prostate cancer. Cancers (Basel).

