# Supplementary material for: Gi/o GPCRs drive the formation of actin-rich tunneling nanotubes in cancer cells via a Gβγ/PKCα/FARP1/Cdc42 axis
Source: J Biol Chem. 2023 Jun 28;299(8):104983. doi: 10.1016/j.jbc.2023.104983 (PMC10374973; doi:10.1016/j.jbc.2023.104983)
Supplement: Supporting Figures S1–S3 and Table S1 [file mmc1.pdf]

SUPPLEMENTAL INFORMATION

Figure S1. Signaling requirement for the formation of TNT-like structures in DU145 prostate cancer cells. Serum starved DU145 cells were treated with 5-oxo-ETE (500 nM), stained with phalloidin rhodamine, and visualized by fluorescence microscopy. *A*, effect of PTX (100 ng/ml, 24 h). *B*, effect of different inhibitors (3 μM gallein, 10 μM GUE1654, 20 μM LY294002, 3 μM gefitinib, 5 μM GF109203X, 5 μM Gö6983, 5 μM Gö6976, 1 h in all cases) on 5-oxo-ETE-induced formation of TNT-like structures. ****$p < 0.0001$ *vs.* no inhibitor.

Figure S2. EGFR transactivation by GPCR ligands in DU145 prostate cancer cells. Western blot for phospho-EGFR and phospho-Akt in response to 5-oxo-ETE (500 nM), LPA (100 nM) or EGF (200 nM) in DU145 cells. A representative experiment is shown.

Figure S3. RNAi-mediated depletion of Rac-GEFs in H295R cells. *A*, H295R cells were transfected with siRNA duplexes for the indicated Rac-GEF or NTC. mRNA levels for each Rac-GEF were determined by Q-PCR. *B*, FARP1 and VAV2 mRNA levels upon silencing with three different siRNA duplexes. Results (mean ± S.E.M.) are expressed as relative to parental (*dotted line*) in each case. *NTC*, non-target control. *$p < 0.05$, **$p < 0.01$, ***$p < 0.001$, ****$p < 0.0001$ *vs.* NTC.

Table S1. List of reagents, antibodies, siRNA duplexes, and Q-PCR probes.

Reagent	Company	Catalog number
Chemicals		
5-oxo-ETE	Cayman	34250
Lysophosphatidic acid (LPA)	Cayman	62215
GF109203X	Tocris	0741
Gö6976	Cayman	13310
Pertussis toxin	Cayman	19546
U73122	Cayman	70740
Gallein	Tocris	3090
LY294002	Tocris	1130
PD98059	Tocris	1213
Docosahexaenoic acid (DHA)	Tocris	3687
Angiotensin II	Tocris	1158
Latrunculin A	Tocris	3973
Cytochalasin D	Tocris	1233
GUE1654	Tocris	4742
ACTH	Tocris	24257
Y-27632	Sigma-Aldrich	Y0503
Epidermal growth factor (EGF)	Sigma-Aldrich	236
Rhodamine phalloidin	Invitrogen	R425
Antibodies		
Anti-PKCα	Cell Signaling	2056S
Anti-PKCδ	Cell Signaling	2058S
Anti-PKCε	Cell Signaling	2683S
Anti-PLCγ1	Cell Signaling	5690S
Anti-Tyr783-PLCγ1	Cell Signaling	2821L/14008S
Anti-EGFR	Cell Signaling	2232S
Anti-Tyr992-EGFR	Cell Signaling	2235L
Anti-Tyr1068-EGFR	Cell Signaling	3777S
Anti-Tyr1101-EGFR	Abcam	Ab76195
Anti-VAV2	Cell Signaling	2848

Anti-Akt	Cell Signaling	4691S
Anti-Ser473-Akt	Cell Signaling	4060L
Anti-Cdc42	Cell Signaling	2466S
Anti-Rac1	Millipore	05-389
Anti-β-actin	Sigma	A5441
Anti-vinculin	Sigma	V4505
Goat Anti-mouse IgG - HRP Conjugate	Bio-Rad	1721011
Goat Anti-rabbit IgG - HRP Conjugate	Bio-Rad	1706515
siRNA duplexes		
PKCα #1	Horizon	J-003523-02
PKCα #2	Horizon	J-003523-02
PKCα #3	Horizon	J-003523-18
PKCδ #1	Horizon	J-003524-07
PKCδ #2	Horizon	J-003524-08
PKCδ #3	Horizon	J-003524-09
PKCε #1	Horizon	J-004653-06
PKCε #2	Horizon	J-004653-07
PKCε #3	Horizon	J-004653-08
PLCβ1 #1	Horizon	J-010280-05
PLCβ1 #2	Horizon	J-010280-06
PLCβ3 #1	Horizon	J-008485-05
PLCβ3 #2	Horizon	J-008485-06
FARP1 #1	Horizon	J-008519-06
FARP1 #2	Horizon	J-008519-07
FARP1 #3	Horizon	J-008519-08
VAV2 #1	Horizon	J-005199-05
VAV2 #2	Horizon	J-005199-06
VAV2 #3	Horizon	J-005199-07
PREX1	Horizon	J-010063-10
PLEKHG2	Horizon	J-023690-06
ARHGEF6	Horizon	J-010231-05
ECT2	Horizon	J-006450-05
RasGRF1	Horizon	J-009323-05
OXER1	Horizon	J-005741-08
Non-target control (NTC)	Horizon	D-001810-10
Q-PCR probes		
PKCα	Dharmacon	Hs00925200
PKCδ	Dharmacon	Hs01090047
PKCε	Dharmacon	Hs00942886
PLCβ1	Dharmacon	Hs01001930
PLCβ3	Dharmacon	Hs01100294
FARP1	Dharmacon	Hs00195010
VAV2	Dharmacon	Hs00610104
PREX1	Dharmacon	Hs00368207
PLECKHG2	Dharmacon	Hs00293943
ARHGEF6	Dharmacon	Hs00374477
ECT2	Dharmacon	Hs00978168
RasGRF1	Dharmacon	Hs01548470
OXER1	Dharmacon	Hs00536961
UBC	Dharmacon	Hs05002522

Figure S1

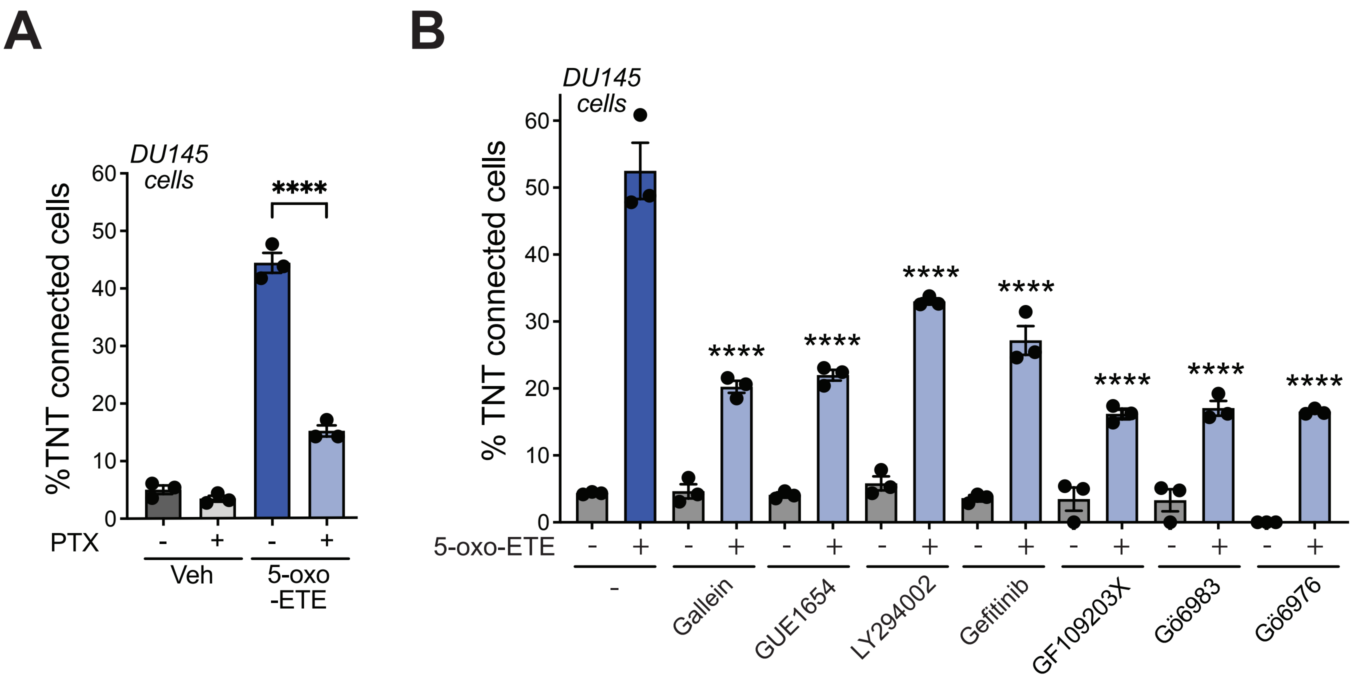
A
DU145 cells
%TNT connected cells
0
10
20
30
40
50
60
****
PTX
-
+
-
+
Veh
5-oxo -ETE
B
DU145 cells
% TNT connected cells
0
10
20
30
40
50
60
****
5-oxo-ETE
-
+
Gallein
GUE1654
LY294002
Gefitinib
GF109203X
Gö6983
Gö6976

Figure S2

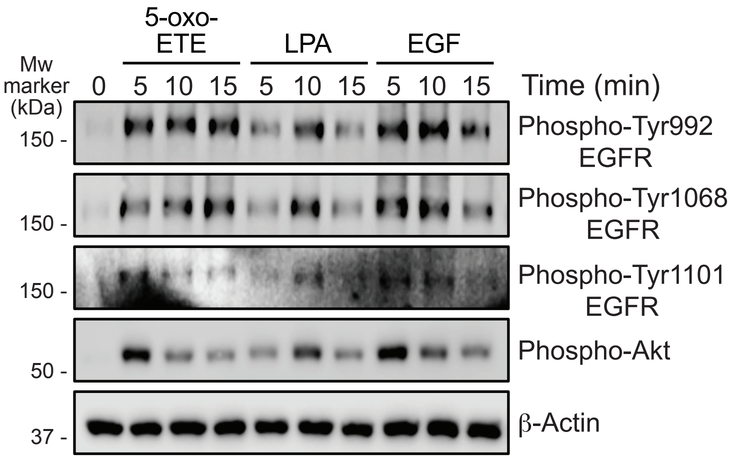
5-oxo-ETE
LPA
EGF
Mw marker (kDa)
0 5 10 15 5 10 15 5 10 15
Time (min)
150 -
Phospho-Tyr992 EGFR
150 -
Phospho-Tyr1068 EGFR
150 -
Phospho-Tyr1101 EGFR
50 -
Phospho-Akt
37 -
β-Actin

Figure S3

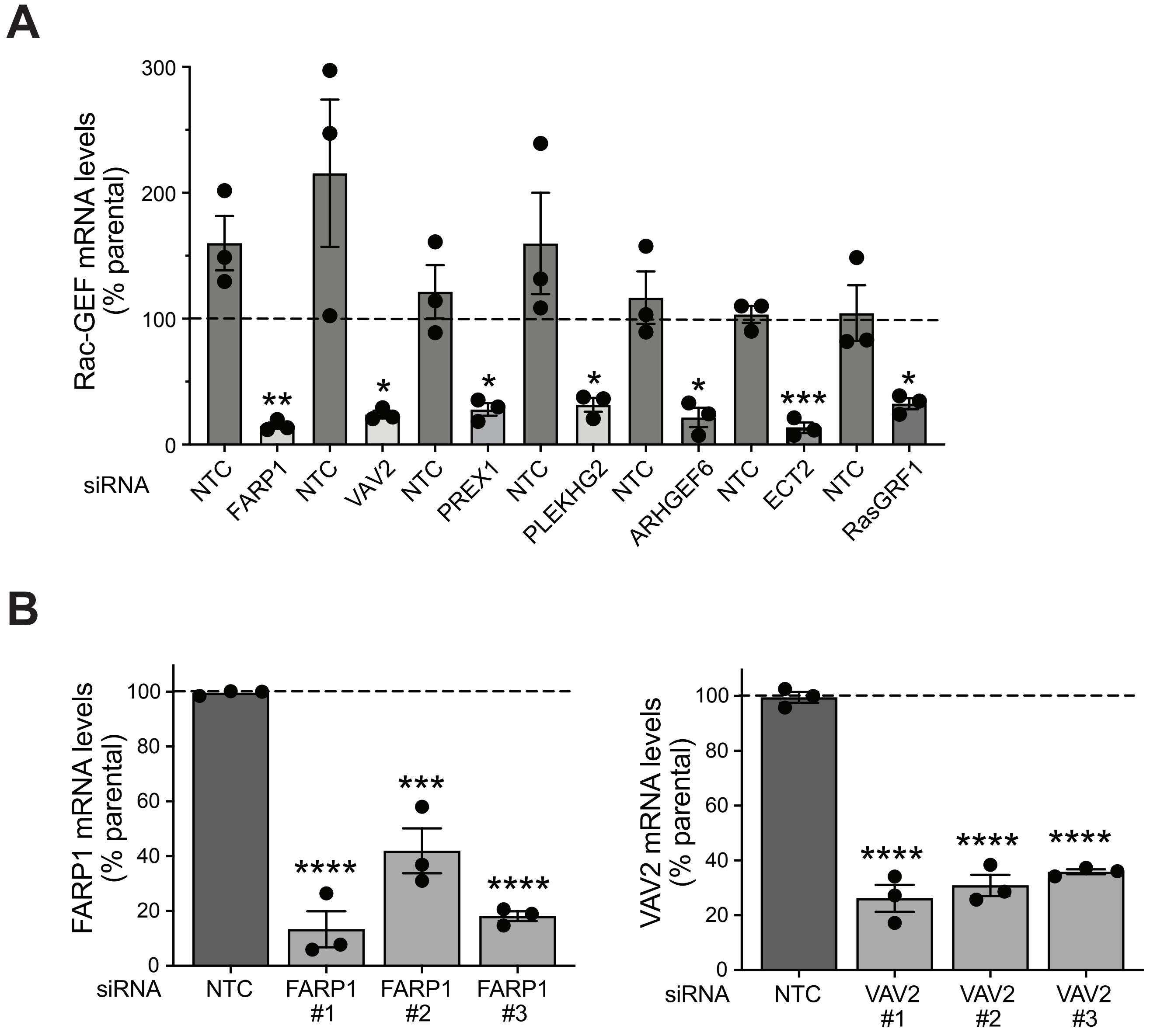
A
Rac-GEF mRNA levels (% parental)
300
200
100
0
siRNA
NTC
FARP1
NTC
VAV2
NTC
PREX1
NTC
PLEKHG2
NTC
ARHGEF6
NTC
ECT2
NTC
RasGRF1
**
*
*
*
*
***
*
B
FARP1 mRNA levels (% parental)
100
80
60
40
20
0
siRNA
NTC
FARP1 #1
FARP1 #2
FARP1 #3
****
***
****
VAV2 mRNA levels (% parental)
100
80
60
40
20
0
siRNA
NTC
VAV2 #1
VAV2 #2
VAV2 #3
****
****
****
